# Change in cognitive function according to cholinesterase inhibitor use and amyloid PET positivity in patients with mild cognitive impairment

**DOI:** 10.1186/s13195-020-00749-5

**Published:** 2021-01-05

**Authors:** Jung-Min Pyun, Nayoung Ryoo, Young Ho Park, SangYun Kim

**Affiliations:** Department of Neurology, Seoul National University College of Medicine and Seoul National University Bundang Hospital, 82, Gumi-ro 173 Beon-gil, Bundang-gu, Seongnam-si, Gyeonggi-do 13620 Republic of Korea

**Keywords:** Mild cognitive impairment, Cholinesterase inhibitor, Amyloid burden, Cognitive change

## Abstract

**Background:**

Cholinesterase inhibitors (ChEIs) are an FDA-approved symptomatic treatment for patients with Alzheimer’s disease (AD). Its efficacy in patients with mild cognitive impairment (MCI), however, is controversial. Nonetheless, ChEIs have often been used in patients with MCI. From the perspective that ChEIs were developed based on the pathomechanism of AD, the effect of ChEIs in MCI patients could be different depending on the amyloid burden. In this retrospective observational study, we aimed to investigate the influence of ChEIs and amyloid burden on cognitive change for 1 year in patients with MCI.

**Methods:**

We included 111 patients with MCI with a Clinical Dementia Rating (CDR) score of 0.5, a 1-year follow-up cognitive assessment, and amyloid positron emission tomography (PET) performed within 6 months before or after the baseline cognitive assessment (73 ChEI users and 38 ChEI non-users) from the Neurocognitive Behavior Center of Seoul National University Bundang Hospital. Additionally, those who had a positive amyloid PET scan more than 6 months before the baseline cognitive assessment and those who had a negative amyloid PET scan more than 6 months after the 1-year follow-up cognitive assessment were also included. Among the total 111 patients, 25 ChEI users and 25 ChEI non-users were matched by baseline Mini-Mental State Examination (MMSE) score, age, educational level, CDR Sum of Boxes, and amyloid PET positivity using propensity score matching. Multiple linear regression analysis was performed to assess the influence of ChEI use and amyloid PET positivity on cognitive change for 1 year. Univariate and multivariate logistic regression analyses were performed to evaluate the association between ChEI use and disease progression to CDR 1 at the 1-year follow-up visit.

**Results:**

ChEI use or non-use was not associated with cognitive change for 1 year. Amyloid PET positivity or negativity did not change this non-association. Furthermore, progression to CDR 1 was related to low baseline MMSE score (OR 0.606, CI 0.381–0.873), but not with ChEI use or non-use, and not with amyloid PET result.

**Conclusion:**

ChEI use or non-use was not related to cognitive change at a 1-year follow-up visit in patients with or without amyloid burden. In addition, ChEI use or non-use could not predict disease progression to CDR 1 at 1-year follow-up visit.

## Background

Cholinesterase inhibitors (ChEIs) are a Food and Drug Administration (FDA)-approved symptomatic treatment for Alzheimer’s disease (AD) and include donepezil, rivastigmine, and galantamine. However, their efficacy in mild cognitive impairment (MCI) is uncertain. According to a recent practice guideline update by Petersen et al., there is no level A evidence regarding ChEI use in MCI, and the recommendations suggest that a physician may choose not to offer ChEIs [1]. Nonetheless, according to a study with the Alzheimer’s Disease Neuroimaging Initiative (ADNI) cohort, 44% of the recruited patients with MCI were treated with ChEIs [2].

A considerable number of studies have previously been conducted to assess the effect of ChEI treatment in patients with MCI [3]. However, participants were included based on a clinical diagnosis of MCI without AD pathology confirmation. Therefore, these study populations might have contained heterogeneous pathologies, which could have led to inconsistent results [3, 4]. The lack of an approved pharmacological treatment for patients with MCI and concern about progression to dementia might lead to the use of ChEIs despite no strong evidence of their efficacy. As ChEIs were developed based on pathological changes in early AD, evaluation of the effect of ChEIs in patients with MCI with AD pathology confirmed by AD biomarkers might provide useful clues to ChEI use regarding the timing of initiating therapy or the indications for treatment.

In this study, we aimed to evaluate the effect of ChEIs on cognition in patients with MCI and their interactions with amyloidopathy.

## Methods

### Participants

A retrospective, longitudinal, and observational study was conducted at Seoul National University Bundang Hospital in the Republic of Korea. We included participants from January 2013 and August 2020 who met the following inclusion criteria: (1) a diagnosis of MCI according to Petersen’s criteria [5], (2) a Clinical Dementia Rating (CDR) score of 0.5 at baseline assessment [6], (3) patients with a 1-year follow-up including a neuropsychological assessment, and (4) those who underwent amyloid positron emission tomography (PET) within 6 months before or after the baseline cognitive assessment. Those who had a positive amyloid PET scan more than 6 months before the baseline cognitive assessment and those who had a negative amyloid PET scan more than 6 months after the 1-year follow-up cognitive assessment were also included. Among a total of 50 patients included for the analysis, 27 participants underwent amyloid PET outside the 6-month window. The time window of the 27 participants ranged from 24 months before to 10 months after the baseline cognitive assessment. The median value was 13 months before the baseline cognitive assessment.

This study design was approved by the Institutional Review Board of Seoul National University Bundang Hospital (B-2006/618-109).

### Data collection

Demographic information such as age, sex, educational level, and *APOE* genotype was collected from the participants. Neuropsychological assessment results at baseline and a 1-year follow-up visit and amyloid PET results were obtained. Use or non-use of ChEIs during the follow-up period after baseline cognitive assessment and the types and dosages of ChEIs prescribed to each user were investigated.

### Cognitive evaluation

We assessed the global cognitive status with the Mini-Mental State Examination (MMSE) [7], dementia severity with CDR Sum of Boxes (CDR SOB), and depressive symptoms with the short form of the Geriatric Depression Scale (GDpS) [8]. Additionally, extensive neuropsychological assessments were performed to evaluate attention, language, verbal and visual memory, visuoconstructive function, and frontal executive function. We used the Digit Span Test for attention [9], the Korean version of the Boston Naming Test for language [10], the Seoul Verbal Learning Test for verbal memory [11], the Rey Complex Figure Test (RCFT) for visuoconstructive function and visual memory [12], the categorical and phonemic fluency test of the Controlled Oral Word Association Test [13], and the Stroop color reading test for executive function [14]. For statistical analysis, MMSE, CDR SOB, and GDpS scores were used as raw scores. The scores for specified neuropsychological tests were converted to standardized scores (*z*-scores), which were adjusted for age, sex, and educational level.

### Amyloid burden

Amyloid burden was evaluated by amyloid PET. [18F]Florbetaben (*n* = 105), [18F]flutemetamol (*n* = 4), and [18F]florbetapir (*n* = 2) were used as ligands. Amyloid status was dichotomized as positive (abnormal) or negative (normal) after visual assessment by one experienced nuclear medicine physician and two neurologists.

### Statistical analysis

To minimize treatment selection bias and the difference in baseline characteristics between ChEI users and non-users, propensity score matching analysis was conducted. Propensity scores were calculated through logistic regression [15] with covariates such as the baseline MMSE score, age, educational level, amyloid PET positivity, and CDR SOB using the *Matchit* packages in R. ChEI users and non-users were paired 1:1 based on these propensity scores with a caliper size of 0.2.

Demographics and clinical characteristics between groups of unmatched and matched sets were compared with Student’s *t* test, the Mann-Whitney *U* test, or the chi-squared test as appropriate. Cognitive assessment at baseline between groups of matched sets was compared using Student’s *t* test or the Mann-Whitney *U* test.

Linear regression analysis was performed to assess the influence of ChEI use or non-use on cognitive change for 1 year. The independent variable was group (ChEI user vs ChEI non-user), and the dependent variable was the difference between the 1-year cognitive test score and the baseline cognitive test score. The GDpS scores were reported as raw scores. For the MMSE analysis, age, sex, and educational level were adjusted. The *z*-scores of the remaining cognitive tests were already adjusted for age, sex, and educational level. Additionally, the multiple linear regression analysis included the amyloid PET scan result as a covariate to evaluate the effect of amyloid burden on the interaction between ChEI use/non-use and cognitive change for 1 year.

Univariate and multivariate logistic regression analyses were performed to investigate the associations among ChEI use/non-use, amyloid burden, and disease progression to CDR 1 at a 1-year follow-up visit.

All statistical analysis was performed using R (version 4.0.0). Statistical significance was set at < 0.05.

## Results

### Clinical characteristics and baseline cognitive function of participants

The total cohort was 60.3% female with a mean age of 71.1 years. Before propensity score matching, 73 patients were ChEI users, and 38 patients were ChEI non-users. ChEI users had significantly lower educational levels, lower baseline MMSE scores, higher disease severity with higher CDR SOB scores, and higher amyloid burden with amyloid PET positivity than ChEI non-users. The propensity score-matched cohort comprised 25 ChEI users and 25 ChEI non-users, and the imbalance in the covariates including the educational level, baseline MMSE score, CDR SOB score, and amyloid PET positivity was alleviated. The covariate differences between groups before and after matching are shown in Table 1. Comparing the baseline cognitive function between the groups in the matched cohort, there was no significant difference (Table 2). MCI subtype of the matched 50 participants was all amnestic MCI.

**Table 1:** Demographics and clinical characteristics of participants before and after propensity score matching

	Before matching		*p* value	After matching		*p* value
	ChEI user (*n* = 73)	ChEI non-user (*n* = 38)		ChEI user (*n* = 25)	ChEI non-user (*n* = 25)	
Age, years	70.8 ± 8.7	71.6 ± 7.5	0.608	71.0 ± 8.8	70.7 ± 8.2	0.908
Female	45 (61.6)	22 (57.9)	0.427	15 (60.0)	15 (60.0)	1.000
Education, years	11.4 ± 4.7	13.2 ± 3.8	0.045	12 [9; 16]	14 [10; 16]	0.889
Amyloid PET positivity	53 (72.6)	17 (44.7)	0.004	14 (56.0)	14 (56.0)	1.000
Baseline MMSE	23.4 ± 2.9	26.3 ± 2.3	0.000	25.0 [24.0; 27.0]	26.0 [24.0; 27.0]	0.709
Baseline CDR SOB	2.5 ± 1.1	1.4 ± 0.8	0.000	1.5 [1.0; 3.0]	1.5 [1.0; 2.0]	0.738
*APOEε*4 carrier*	33 (62.3)	11 (42.3)	0.076	15 (60.0)	5 (31.2)	0.012
Cholinesterase inhibitor						
Donepezil	70 (95.9)			24 (0.96)		
Rivastigmine	3 (4.1)			1 (0.04)		

Data are presented as the mean ± standard deviation, median [interquartile range], or *n* (%)

*There were 35 missing values among 111 patients from the unmatched cohort and 15 missing values among 50 patients from the matched cohort

**Table 2:** Comparison of cognitive function at baseline between ChEI users and non-users

	ChEI user (*n* = 25)	ChEI non-user (*n* = 25)	*p* value
Digit Span Forward Test	0.02 ± 0.84	− 0.29 ± 0.81	0.223
Digit Span Backward Test	− 0.55 [− 0.91; 0.26]	− 0.64 [− 1.14; − 0.15]	0.219
Korean Boston Naming Test	− 0.76 [− 1.28; 0.55]	− 0.75 [− 1.05; 0.17]	0.854
SVLT Immediate recall	− 1.03 [− 1.41; − 0.50]	− 1.26 [− 1.35; − 1.06]	0.503
SVLT Delayed recall	− 1.45 [− 1.45; − 1.24]	− 1.32 [− 1.45; − 0.99]	0.394
SVLT Recognition	− 1.15 [− 1.45; − 0.42]	− 1.12 [− 1.43; − 0.49]	0.611
RCFT Copy	− 1.06 [− 1.44; − 0.37]	− 0.96 [− 1.43; 0.15]	0.930
RCFT Delayed recall	− 1.36 [− 1.44; − 1.07]	− 1.14 [− 1.43; − 0.76]	0.154
RCFT Recognition	− 1.04 [− 1.40; − 0.65]	− 0.45 [− 1.42; − 0.03]	0.214
Categorical fluency	− 0.80 [− 1.04; − 0.28]	− 0.54 [− 1.25; − 0.33]	0.808
Phonemic fluency	− 0.87 [− 1.14; 0.25]	− 0.84 [− 1.22; 0.26]	0.340
Stroop Colors Test	− 0.50 [− 1.32; 0.27]	− 0.84 [− 1.44; 0.20]	0.340
Geriatric Depression Scale	4.00 [2.00; 6.00]	5.00 [1.00; 8.00]	0.792

Data are presented as the mean ± standard deviation or median [interquartile range]. Raw scores were used for the Geriatric Depression Scale, and *z*-scores were used for the rest of the neuropsychological tests

### Effect of ChEIs on cognition change at 1-year follow-up and amyloid burden

Univariate linear regression analysis was performed to evaluate the effect of ChEIs on cognitive change at a 1-year follow-up visit. ChEI use was not significantly associated with cognitive change at 1 year. Adding the amyloid PET results as a covariate, multivariate linear regression analysis was performed to assess the influence of amyloid burden on cognitive change. Amyloid PET positivity did not alter the non-association between ChEI use and cognitive change (Table 3).

**Table 3:** Change in cognitive function after 1 year according to group and association with amyloid burden as analyzed by linear regression analysis

Change in	*β* (SE), *p* value	*β* (SE), *p* value Including amyloid PET result
MMSE	0.486 (0.720), 0.503	0.488 (0.718), 0.499
CDR SOB	− 0.460 (0.367), 0.217	− 0.460 (0.371), 0.221
Geriatric Depression Scale	− 0.810 (1.074), 0.455	− 0.821 (1.084), 0.453
Digit Span Forward Test	− 0.024 (0.198), 0.901	− 0.015 (0.199), 0.938
Digit Span Backward Test	0.004 (0.233), 0.985	0.030 (0.227), 0.894
Korean Boston Naming Test	0.088 (0.221), 0.692	0.088 (0.223), 0.694
SVLT Immediate recall	0.024 (0.171), 0.887	0.024 (0.173), 0.888
SVLT Delayed recall	− 0.162 (0.127), 0.208	− 0.162 (0.127), 0.210
SVLT Recognition	0.136 (0.206), 0.511	0.136 (0.207), 0.514
RCFT Copy	0.110 (0.235), 0.642	0.110 (0.236), 0.644
RCFT Delayed recall	− 0.119 (0.127), 0.355	− 0.116 (0.128), 0.371
RCFT Recognition	0.385 (0.284), 0.183	0.378 (0.288), 0.198
Categorical fluency	0.215 (0.161), 0.189	0.215 (0.161), 0.189
Phonemic fluency	− 0.132 (0.209), 0.530	− 0.132 (0.196), 0.503
Stroop Colors Test	0.153 (0.174), 0.384	0.153 (0.174), 0.382

The dependent variable was the change in cognitive test scores over 1 year. The independent variable was the group (ChEI user vs ChEI non-user). The amyloid PET result was adjusted. Age, sex, and educational level were also adjusted in the MMSE analysis. *β* values (standard error, SE) of the dependent variable are presented by group. Raw scores were used for the CDR SOB and Geriatric Depression Scale, and *z*-scores were used for the rest of the neuropsychological tests

### Predictors of progression to CDR 1 at 1-year follow-up

Univariate and multivariate logistic regression analysis showed that ChEI use was not related to disease progression to CDR 1 at the 1-year follow-up visit. A low MMSE score at baseline could predict progression to CDR 1 in the univariate and multivariate analyses (Table 4).

**Table 4:** Predictors of progression to CDR 1 at 1-year follow-up using logistic regression analysis

	Univariate OR (95% CI), *p* value	Multivariate OR (95% CI), *p* value
Age	1.018 (0.933–1.115), 0.685	1.030 (0.934–1.146), 0.549
Female	2.739 (0.577–19.913), 0.242	2.089 (0.314–1.805), 0.455
Education	0.929 (0.784–1.108), 0.395	1.081 (0.885–1.357), 0.453
Amyloid PET positivity	1.727 (0.397–9.072), 0.479	3.925 (0.676–34.315), 0.157
Baseline MMSE	0.662 (0.451–0.895), 0.015	0.606 (0.381–0.873), 0.015
ChEI use	1.312 (0.305–5.975), 0.713	1.120 (0.204–6.238), 0.892

## Discussion

This study demonstrated that ChEI use was not associated with cognitive change during a 1-year follow-up period, and amyloid PET positivity did not alter this non-association between ChEI use and cognitive change. Additionally, ChEI use or non-use could not predict disease progression to CDR 1 at the 1-year follow-up visit. A low baseline MMSE score, however, could predict disease progression.

Previous studies regarding ChEI use in patients with MCI have not shown convincing results. A trial with donepezil or placebo reported that the donepezil group had a lower rate of progression to dementia during the first year; however, at the endpoint of 3 years, the rate was not lower than that in the placebo group [16]. Other trials with donepezil showed statistically significant changes in Alzheimer’s Disease Assessment Scale-Cognitive Subscale (ADAS-cog) scores [17, 18]; however, its clinical relevance was questioned [1]. Rivastigmine and galantamine did not demonstrate a benefit for the progression to AD [19, 20]. The lack of a favorable effect of ChEIs on cognition found in our study was similar to the results from the previous literature.

ChEI use in AD is based on the cholinergic hypothesis, which embodies progressive loss of limbic and neocortical cholinergic innervation in AD [21]. In particular, as the source of cortical cholinergic innervation, the basal forebrain neurons are the most vulnerable areas in which the pathological changes of AD such as amyloid plaques and neurofibrillary tangles are observed [22]. In the prodromal stage of AD, the basal forebrain neurons have been considered dysregulated, albeit viable, implying a possibility of intervention [23].

A longitudinal study using functional magnetic resonance imaging showed that patients with MCI treated with donepezil showed activation in the ventrolateral prefrontal cortex [24]. Patients with prodromal AD under donepezil treatment showed a reduced rate of hippocampal atrophy compared with those under treatment with placebo; however, no significant cognitive differences were noted [25]. These results might suggest that ChEI use in patients with MCI could affect pathophysiological changes but not symptomatic changes. This could be due to the short follow-up period of the studies or the relatively preserved cognitive reserve of the MCI population, which could also be possible in our study participants with high baseline MMSE score and high educational levels. A longer follow-up duration might be required to observe cognitive differences.

Interaction of cholinesterase and amyloid metabolism has not been clearly elucidated. Cholinesterase could promote amyloid-ß aggregation [26], and amyloid-ß also increases cholinesterase levels [27]. In addition to having a direct link with amyloidopathy, ChEIs could also protect neurons from amyloid-ß-induced injury and attenuate cytokine release from microglia, showing an anti-inflammatory effect [28]. Based on this evidence, the effect of ChEI use in AD could be partially contributed to by other pathological processes such as neuroinflammatory pathways. The lack of influence of amyloid positivity or negativity on the relation of ChEI use to cognitive change in our study might be partially explained by this evidence.

Additionally, several studies have evaluated the association of *APOE* genotypes and cognitive response to ChEIs in AD. The results are mixed and could not demonstrate a significant influence of *APOE* genotype on ChEI response [29–31]. Although the interaction of the *APOE* ε4 allele and cholinergic pathways in the basal forebrain, including its involvement of the amyloid pathway, has not been elucidated clearly, a recent animal study revealed that a compensatory cholinergic sprouting for hippocampal dysfunction due to neuronal loss in the entorhinal cortex is highly sensitive to the presence of the *APOE* ε4 allele [32]. This result could enlighten further studies to understand ChEI response to the cholinergic system with initial AD-related pathological changes in MCI.

To our knowledge, this is the first study to investigate the effect of ChEIs on cognitive change and their interaction with amyloid burden. A cohort of patients with MCI with confirmed AD biomarkers would be a homogenous and appropriate population to evaluate the effect of ChEIs, which could produce results with great significance. Our study and a further validation study with a larger sample size and longer follow-up period might provide a potential for improving ChEIs as treatment strategies for patients with MCI.

### Limitations

The limitations of the current study are the small sample size and short follow-up period. Although the sample size was reduced through propensity score matching, this analytic method could lead to reliable results by reducing the selection bias of observational studies. Furthermore, Petersen et al. reported that the estimated annual rate of MCI conversion to AD ranges between 10 and 15%, and patients with amnestic MCI are prone to progress to AD [5]. A systematic review also demonstrated that a conversion rate over 1 year spanned from 10.2 to 33.6% and over 2 years spanned from 9.8 to 36.3% in clinical samples [33]. Given the short follow-up period in our study, a study with a bigger cohort of MCI with longer follow-up time will be needed. The current result could be considered as a preliminary study for further research.

## Conclusions

In summary, ChEI use was not associated with cognitive change during a 1-year follow-up period in MCI, and amyloid PET positivity did not affect this result. Furthermore, ChEI use or non-use could not predict disease progression to CDR 1 at the 1-year follow-up visit. This might suggest a clue of strategies regarding use of ChEIs in patients with MCI.

## Data Availability

The study data are not publicly available for download, but might be retrieved from corresponding author professor SangYun Kim.

## Abbreviations

AD: Alzheimer’s disease
ADAS-cog: Alzheimer’s Disease Assessment Scale-Cognitive Subscale
ADNI: Alzheimer’s Disease Neuroimaging Initiative
CDR: Clinical Dementia Rating
CDR SOB: Clinical Dementia Rating Sum of Boxes
ChEI: Cholinesterase inhibitor
CI: Confidence interval
FDA: Food and Drug Administration
GDpS: Geriatric Depression Scale
MCI: Mild Cognitive Impairment
MMSE: Mini-Mental State Examination
OR: Odds ratio
PET: Positron emission tomography
RCFT: Rey Complex Figure Test
SVLT: Seoul Verbal Learning Test
